# Green and Rapid Synthesis of Anticancerous Silver Nanoparticles by *Saccharomyces boulardii* and Insight into Mechanism of Nanoparticle Synthesis

**DOI:** 10.1155/2013/872940

**Published:** 2013-11-05

**Authors:** Abhishek Kaler, Sanyog Jain, Uttam Chand Banerjee

**Affiliations:** ^1^Department of Pharmaceutical Technology (Biotechnology), National Institute of Pharmaceutical Education and Research, Sector-67, SAS Nagar, Punjab 160062, India; ^2^Centre for Pharmaceutical Nanotechnology, Department of Pharmaceutics, National Institute of Pharmaceutical Education and Research, Sector-67, SAS Nagar, Punjab 160062, India

## Abstract

Rapidly developing field of nanobiotechnology dealing with metallic nanoparticle (MNP) synthesis is primarily lacking control over size, shape, dispersity, yield, and reaction time. Present work describes an ecofriendly method for the synthesis of silver nanoparticles (AgNPs) by cell free extract (CFE) of *Saccharomyces boulardii*. Parameters such as culture age (stationary phase growth), cell mass concentration (400 mg/mL), temperature (35°C), and reaction time (4 h), have been optimized to exercise a control over the yield of nanoparticles and their properties. Nanoparticle (NP) formation was confirmed by UV-Vis spectroscopy, elemental composition by EDX (energy dispersive X-rays) analysis, and size and shape by transmission electron microscopy. Synthesized nanoparticles had the size range of 3–10 nm with high negative zeta potential (−31 mV) indicating excellent stability. Role of proteins/peptides in NP formation and their stability were also elucidated. Finally, anticancer activity of silver nanoparticles as compared to silver ions was determined on breast cancer cell lines.

## 1. Introduction

Classical physicochemical synthetic procedures of metal nanoparticles suffer from drawbacks such as polydispersity, low yield [1], and use of toxic reagents [2]. Traditional role of nanosilver as an antimicrobial agent [3] and an anti-inflammatory agent [4] is well known. Recently, nanosilver has found application in electrical batteries [5], as optical receptors in solar batteries [6], biolabelling [7], and in cancer treatment [8]. Considering such wide range of applications of AgNPs, there is a need to develop clean, nontoxic, and ecofriendly synthetic approach. Microorganisms (bacteria, fungi, yeast, etc.) are known to produce inorganic materials synthetic protocols for nanoparticle production that utilizes ecofriendly nanofactories. There are numerous reports available on the formation of various MNPs by microorganisms from their corresponding salts [9–12]. Microbial systems are mainly advantageous in terms of ecofriendliness. However, major drawbacks associated with these systems are longer reaction time, tedious purification steps, higher size of all nanoparticles, and poor understanding of mechanism. Microorganisms can synthesize nanoparticles both extracellularly and intracellularly, the former being advantageous as it minimizes the number of steps in downstream processing. The present work describes a method that utilized cell free extract of *S. boulardii* for the synthesis of silver nanoparticle in pure form. Furthermore, to gain control on the shape and size of the nanoparticle and to increase its yield, the effect of the physicochemical environment for CFE production has been checked and process parameters were optimized. Longer reaction time was shortened by concentrating CFE using freeze dryer. Finally, role of proteins and/or peptides in NP formation and their stabilization were established by inactivating the cellular machinery at various growth levels and by FTIR technique. Many metals including platinum [13], copper [14], and selenium [15], are being proven to be useful as anticancer agents. The nano form of silver was also compared to its ionic form against breast cancer cell lines for its enhanced activity.

## 2. Materials and Methods

### 2.1. Chemicals

Silver nitrate (99.99%) was purchased from Sigma-Aldrich (Steinheim, Germany). Unless specified, different salts and media components were purchased from HiMedia (Mumbai, India).

### 2.2. Microorganism and Culture Conditions

The yeast *Saccharomyces boulardii*, a probiotic strain, was purchased from a local market. Seed culture was developed by inoculating a single colony of *S. boulardii* into growth medium (20 mL) containing peptone (3 g/L), yeast extract (3 g/L), and dextrose (10 g/L). The culture was incubated at 35°C for 24 h at 200 rpm after adjusting the pH to 6.5. Inoculum (2% v/v) was transferred to the production medium of the same composition and culture was grown for 48 h at 35°C and 200 rpm. After sufficient growth, cell mass was harvested by centrifugation at 10,000 ×g for 10 min. Cell mass was washed thrice with deionised water to remove remaining media components from cell surface and used for CFE preparation.

### 2.3. Preparation of Cell Free Extract (CFE)

Harvested *S. boulardii* cells were resuspended in deionised water to obtain desired cell mass concentration. This cell suspension was incubated at 35°C for 36 h at 200 rpm. After 36 h, cell suspension was centrifuged at 10,000 ×g for 10 min and supernatant was collected. Cell free extract was also lyophilized to powder form and resuspended in minimum volume of deionised water. Silver nitrate was added and reaction was allowed to proceed in dark for specific period of time with periodic sampling.

### 2.4. Synthesis and Characterization of Silver Nanoparticles

Silver nitrate (1 mM) was added to cell free extract (10 mL) (prepared from cells harvested after 48 h of growth and having 100 mg/mL cell mass concentration) in a conical flask of 50 mL capacity. The reaction was allowed to proceed at 35°C (200 rpm) in the dark environment for 72 h. Two controls were used simultaneously (one having only silver nitrate in deionised water and other having only CFE without silver nitrate). The reaction mixture was periodically analyzed using U-3010 UV-Vis spectrophotometer (Hitachi) in the range of 200–800 nm unless the further increase in absorbance ceased. Synthesized Ag-nanoparticles were visualized by transmission electron microscopy (Hitachi, H-7500 instrument at a voltage of 80 kV). Silver nanoparticle film was formed on carbon coated copper grids and the micrograph of the sample was recorded. For the determination of particle potential, dynamic light scattering analysis was done using zeta sizer (Malvern Zeta Sizer, Nano-ZS). Biochemical nature of the capping agent present on surface of silver nanoparticles was determined using Fourier transform infrared spectroscope (FTIR). Energy-dispersive X-ray (EDX) spectra were obtained on a JEOL JEM-2010F microscope operated at 200 kV.

### 2.5. Bradford Assay for Protein Determination

The protein content was determined by Bradford assay, using BSA as a standard [16]. The absorbance was measured on MultiSkan Thermo 96-well plate reader at 595 nm. All the experiments were performed in triplicates on 96-well plates. 

## 3. Optimization of Physicochemical Parameters for the Synthesis of Silver Nanoparticles

### 3.1. Cell Age and Its Concentration

To determine optimum cell age for the nanoparticle synthesis, yeast cells were harvested at different phases of their growth (log phase, early stationary phase, and late stationary phase). Harvested cells from different growth phases were then resuspended in deionised water and cell free extract was prepared as mentioned above. This cell free extract was used to synthesize silver nanoparticles in dark environment. After harvesting, wet cell mass was weighed correctly and then resuspended in the fixed volume of deionised water to give a specific concentration. Different concentrations of cells (25–500 mg/mL) were incubated in deionised water at 35°C, 200 rpm for 48 h followed by centrifugation at 10,000 ×g for 10 min. Upon addition of silver nitrate (final concentration 1 mM) to the cell free extract, reaction was carried out at 35°C, 200 rpm in dark environment.

### 3.2. Reaction Temperature

Effect of temperature on the nanoparticle synthesis was studied by incubating the cells in deionised water at various temperatures (20, 25, 30, 35, 40, and 45°C) for 48 h. Cell free extract was then collected by centrifugation at 10,000 ×g for 10 min. Silver nitrate was added to the cell free extract to a final concentration of 1 mM and reaction was allowed to proceed at the corresponding temperature and (200) rpm in dark environment.

### 3.3. Reaction pH

Hydrogen ion concentration of cell free extract was adjusted to acidic and basic values (pH range from 3 to 11) by acetic acid and sodium hydroxide, respectively. Reaction mixture containing CFE of various pH values and silver nitrate was incubated at 35°C for 48 h at 200 rpm in dark condition.

### 3.4. Reaction Time

Effect of reaction time was studied by incubating the reaction mixture at optimized condition up to 72 h. Samples were taken at a periodic interval and analyzed by UV-Vis spectrophotometer.

## 4. Use of Cellular Machinery in Nanoparticle Synthesis

Role of various cellular preparations was evaluated to have an insight into the process of nanoparticle biosynthesis. Initially, nanoparticle synthesis was carried out by resuspending whole cells in deionised water and using it as reaction mixture. The process required the disintegration of cells to recover nanoparticles, making downstream process difficult. To evaluate the involvement of cellular components in nanoparticle synthesis, at the same time, cells after sufficient growth were heat-killed in growth medium by autoclaving, centrifuged out, and resuspended in deionised water. Separate set of experiments were carried out on the harvested cells (alive) that were resuspended in deionised water and heat-killed by boiling them for half an hour in water bath. To all the preparations, silver nitrate was added to a final concentration of 1 mM and reaction was allowed for 72 h (200 rpm) in dark conditions. It may also be mentioned that supernatant from microbial growth did not perform the reaction.

Cell free extract was developed at later stages with the purpose of making the reaction system extracellular. To establish the correlation between metabolic machineries of whole cells and CFE, further experiments were performed on cell free extract. Cell free extract was separated into three fractions. First fraction was boiled to denature proteins/enzymes. Second fraction was kept within dialysis membrane (10 KDa) and placed in a beaker containing silver salt solution. Third fraction was a control reaction containing cell free extract and silver nitrate. To all the fractions, silver nitrate solution (1 mM) was added and reaction was allowed to proceed for 72 h.

In another set of experiments, further hint about nature of enzyme was explored. Silver nanoparticle formation is a reduction process and might have involved a reducing enzyme. That reducing enzyme in most of the cases depends on the cofactors. To check whether protein (that could be enzyme also) involved in reduction process is dependent on cofactor, exogenous cofactor (NADH) was added to the reaction mixture.

## 5. Cytotoxicity Studies of Silver Nanoparticles

Cytotoxicity study was done by a method developed by Skehan et al. [17]. Briefly, MCF-7 cells were harvested in log phase using trypsin (0.05% trypsin, 0.02% EDTA, in PBS), counted using a haemocytometer and 1 × 10^4^ cells/well in an aliquot of 100 *μ*L were seeded in 96-wells cell culture plates. The cells were incubated for 24 h (at 37°C in an atmosphere of 5% CO_2_ and 95% relative humidity in a CO_2_ incubator) to adhere. Test materials (100 *μ*L/well) were then added to the wells. The plates were further incubated for 24 h in the CO_2_ incubator. The cells were then fixed by gently layering trichloroacetic acid (50 *μ*L/well, 50% w/v) on top of the medium in all the wells and incubated at 4°C for 1 h. The plates were washed five times with distilled water and air-dried. Cell growth was measured by staining with sulforhodamine B dye (0.4% w/v in 1% acetic acid, 100 *μ*L/well). The unbound dye was washed 3–5 times with 1% acetic acid and plates were air dried. The adsorbed dye was dissolved in Tris-buffer (100 *μ*L/well, 0.01 M, pH 10.4) and plates were gently shaken for 10 min on a mechanical shaker. The optical density (OD) was recorded using a 96-well plate reader at 540 nm. Growth inhibition was calculated by the following formula:

(1) %  Growth  inhibition  =100−[ODtest  sample−ODblankODcontrol−ODblank]×100.

## 6. Results and Discussion

**Figure 1:**
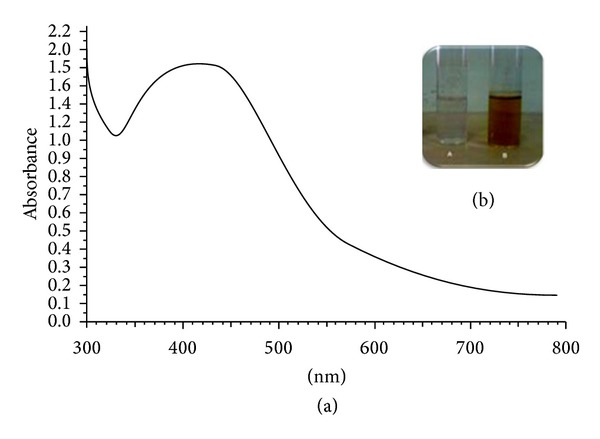
(a) UV-Vis spectrum of CFE in deionised water reacted with silver nitrate (1 mM) for 72 h. (b) Vials containing CFE of *S. boulardii* in deionised water without silver nitrate (Vial A) and with silver nitrate (1 mM) (Vial B) after 72 h incubation at 30°C, 200 rpm.

First step was to develop a catalyst system that can be used to synthesize stable nanoparticles in simple way while minimizing down streaming process. In an attempt to device a greener and less tedious methodology for the AgNPs formation, cell free extract was prepared. Factor(s) responsible for the reduction of metal salt may include but not limited to proteins, enzymes, and amino acid. Keeping in view these factors, cells were harvested from media and resuspended in demineralised water to prepare cell free extract. Formation of AgNPs was accompanied by colour change of the solution from colourless to dark brown which showed absorption in the visible range of electromagnetic radiation [12]. The formation of silver nanoparticles in the solution was confirmed by UV-Vis spectral analysis.  Figure 1(a) shows UV-Vis spectrum of reaction mixture containing CFE and silver nitrate (1 mM) incubated for 72 h. Strong absorption at 420 nm confirms the formation of silver nanoparticles in solution. Parallel control experiments containing only CFE (without AgNO_3_) and AgNO_3_ solution (without CFE) did not show any absorption at ~420 nm.  Figure 1(b) shows two vials, one containing only CFE of *S. boulardii* (Vial A) and another containing reaction mixture of CFE and silver nitrate (Vial B), both incubated for 72 h at 35°C (200 rpm). Colour of the solution in Vial B changed from colourless to dark brown gradually, while Vial A did not exhibit any colour change. 

**Figure 2:**
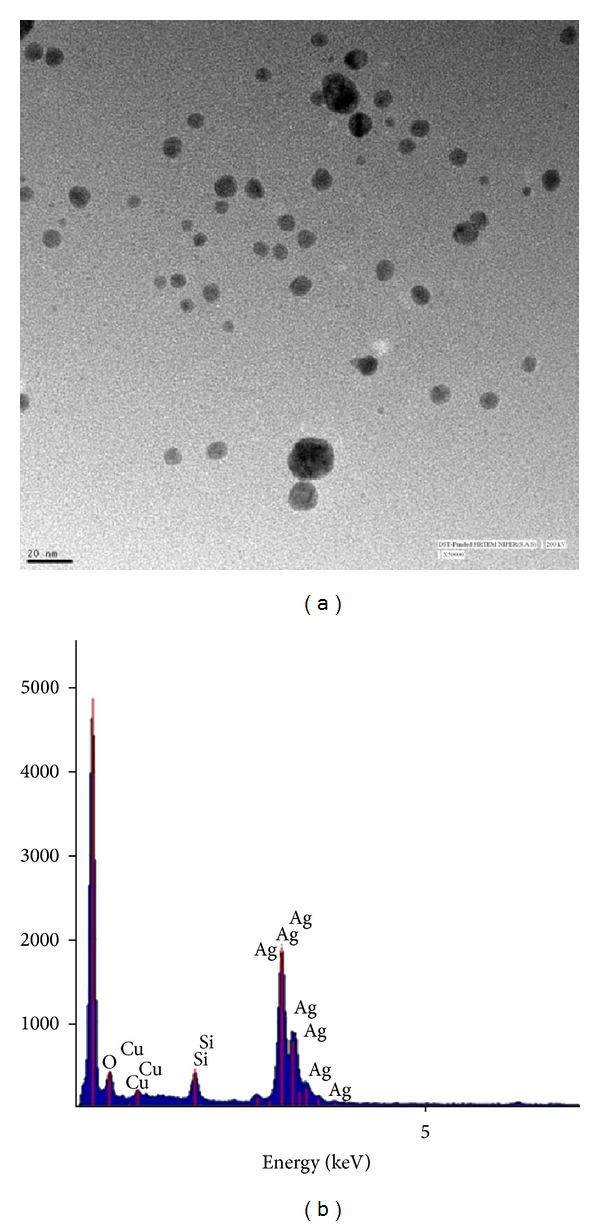
(a) TEM image and (b) EDX image of silver nanoparticles synthesised by CFE of *S. boulardii *in deionised water with silver nitrate (1 mM) after 12 h incubation at 37°C, 200 rpm.

Figure 2(a) shows a representative TEM image of monodispersed AgNPs with size range of 3–10 nm. Previous investigators have reported the synthesis of Ag-nanoparticles having particle size of around 60 nm [18]. Dynamic light scattering analysis of the colloidal solution further confirmed monodispersity of the synthesized particles (PDI of 0.34). Zeta potential value of −31 mV on nanoparticles, indicates the presence of negatively charged molecules on the particles, which prevents them from agglomeration. Elemental composition of synthesized nanoparticle was determined by EDX spectra that showed strong signal of Ag^0^ with high weight percentage of Ag (Figure 2(b)).

FTIR analysis carried out on a drop-coated film of the silver nanoparticle that showed the presence of two bands of N–H and carbonyl at 3353 cm^−1^ and 1650 cm^−1^ due to the stretching vibrational frequency of amide bond, respectively. Bands at 1403 cm^−1^ are due to the side chain of amino acid such as CN stretch of amino group or carboxy group. Peak at 1049 cm^−1^ have aroused due to the aliphatic C–O stretching vibration (serine C–OH). All these observations indicate the involvement of proteins in the reduction and capping process. Previous reports have supported this fact where metal-protein interaction has been shown [19, 20]. Ahmad et al. [12] and Kalimuthu et al. [21] have reported the involvement of a nitrate reductase in a NADH dependent reduction of silver ion to metallic silver. *Fusarium oxysporum* is known to carryout a similar reduction through a nitrate dependent reductase which acts in shuttle with quinone [22]. 

Studies by Balaji et al. [23] have proved the role of carbonyl groups from amino acid residues and peptides of proteins to have strong affinity to Ag. This protein works by forming a protective coat on the nanoparticle surface preventing its aggregation and thus in stabilisation.

**Figure 3:**
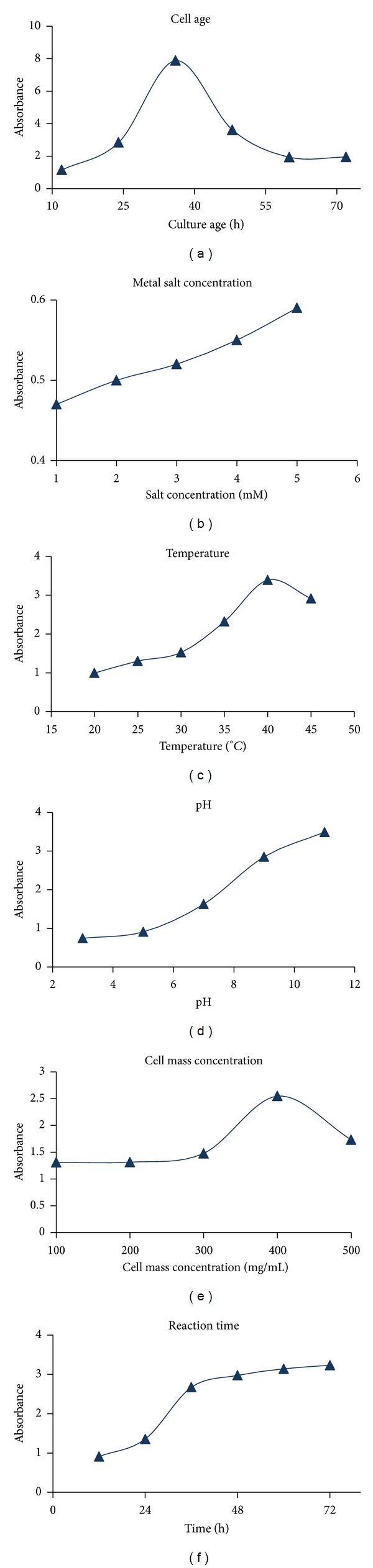
Optimisation of various physicochemical parameters for the synthesis of silver nanoparticles by cell free extract of* Saccharomyces boulardii*.

Preliminary results established CFE as a suitable replacement of whole cells for Ag-nanoparticle synthesis. Therefore, conditions affecting CFE generation were evaluated and various reaction parameters were optimized. Metabolic activities of the cells vary with its age and CFE prepared from these cells will vary in composition as well as in the corresponding activity.  Figure 3(a) shows the varying effect on nanoparticle synthesis of CFE prepared from the cells harvested during different phases of growth (log phase, early stationary phase, and late stationary phase). CFE obtained from early stationary phase cells was the most efficient for nanoparticle synthesis. Furthermore, incubation period of 36 h gave better results as longer period of incubation of the cells at 40°C might have led to the denaturation of the proteins.

Silver ions beyond a certain limit can be toxic to proteins as they can cause precipitation. To prevent precipitation, ionic forms are transformed to nanoparticles by proteins. To determine maximum tolerable limit of CFE proteins to silver ions, different concentrations of silver nitrate were added.  Figure 3(b) shows that no significant increase in absorption was there with the increase in silver nitrate concentration from 1 to 5 mM. That might be due to higher toxicity of silver ions at this concentration. Subsequent experiments were carried out with 1 mM salt concentration. Temperature can affect the rate as well as extent of protein/peptide release into the surrounding medium during CFE formation. Secondly, rate of reaction can be controlled by varying the reaction temperature. The effect of temperature on CFE formation was studied by incubating cell mass at different temperatures.  Figure 3(c) shows the UV-Vis spectra of the Ag-nanoparticles formed with the CFE prepared at different temperatures. As the temperature of CFE preparation was increased the concentration of nanoparticles was found to increase. Optimum temperature for CFE formation was found to be 40°C beyond which there was again a fall in the absorbance of synthesized nanoparticles. Plausible explanation could be that at higher temperature the cells might have released more reducing agent due to thermal shock. Still at higher temperature (>40°C), protein denaturation might take place leading to fall in nanoparticle synthesis. Gericke and Pinches [24] also found similar effect of temperature for the Au-nanoparticle synthesis using *Verticillium luteoalbum *whole cells. 

Nanoparticle formation starts following the process of crystallisation process which depends on factors like solubility of substrate and its supersaturation. Effect of pH on solubility of substrate is known and change in pH can affect the nanoparticle synthesis as well as morphology [25]. It was observed in the present study that nanoparticle formation was higher at higher pH value. The increased synthesis of nanoparticles at higher pH is however nullified by the formation of aggregates at this pH. Optimum pH which showed significant yield without aggregation was selected to be 7 for further studies (Figure 3(d)). Concentration of reducing agent in CFE can be varied by varying the cell concentration used for preparation of CFE.  Figure 3(e) summarizes the effect of CFE prepared using different cell mass concentrations on the nanoparticle formation. Average size of the particles decreased and yield increased with increase in reductant concentration in the CFE (Figure 3(e)). Smallest particle size was obtained with the CFE prepared from 400 mg/mL cell mass concentration. Also, the UV-Vis spectra showed the highest absorbance by nanoparticles formed by the CFE prepared from the increased amount of cell mass. Higher ratio of reducing agent to substrate accelerated the reduction of the Ag^+^ to Ag^0^ immediately followed by capping by the capping agents preventing from aggregation. Recent literature on metallic nanoparticle synthesis has demonstrated the use of whole cells for both extracellular as well as intracellular nanoparticle formation [26, 27].

Time taken to complete nanoparticle synthesis is an important parameter from an industrial point of view. Moreover, aggregate formation of nanoparticles, a sign of instability, is also a problem when longer reaction time is desired. After optimization of the above mentioned parameters, it was found that nanoparticle synthesis was completed after 36 h as compared to initial 72 h of reaction in unoptimized conditions (Figure 3(f)). 

Longer time taken to synthesize silver nanoparticle can be attributed to two factors. First is low redox potential of silver and second being dilute CFE. To overcome this problem, CFE was concentrated by lyophilisation technique. Lyophilisation of proteins can have deleterious effect on their properties and activity [28]. In the present case, however, it did not pose any such problem. CFE after concentration to powder form was resuspended in minimum volume of deionised water and subjected to reaction with silver nitrate as mentioned above. This step significantly reduced the reaction time from 36 h to 4 h with reaction that started immediately after addition of substrate.

**Figure 4:**
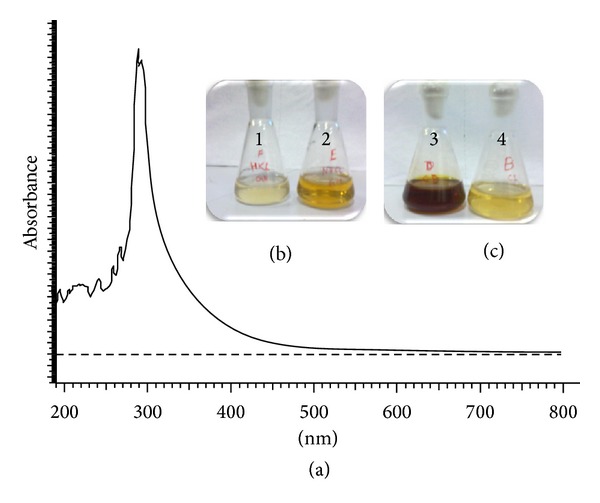
(a) UV-Vis spectra of cell free extract prepared from *Saccharomyces boulardii* in deionised water. (b) Colour comparison between reaction mixtures containing cells killed (1) in production media and control (2). (c) Colour comparison between reaction mixtures containing cofactors (3) and control (4).

To establish the role of cellular machinery in nanoparticle synthesis, yeast cells were subjected to heat treatment at various stages of growth. The cells were grown upto stationary phase and then killed and prepared for CFE. Heat-killed cells during growth phase (Figure 4(b)) were not able to produce AgNPs when treated with silver nitrate. This simple experimentation shows that nanoparticle synthesis essentially depends upon the active metabolites obtained from the native cells and not from the heat-killed cells. The results of Bradford assay showed the presence of protein in the cell free extract. It was assumed that small proteins/peptides are extracted out of cell due to osmotic shock during CFE formation. UV-Vis spectra of CFE (Figure 4(a)) showed a strong absorption at 280 nm which may be attributed to the aromatic residues (tyrosine and tryptophan) present in the protein or peptide. 

The above assumption was strengthened by boiling a fraction of CFE and checking this fraction for its ability to synthesise AgNPs. This fraction did not synthesize nanoparticles and this can be attributed to denaturation of proteins present in CFE. Involvement of macromolecule like protein is further confirmed by placing a barrier (dialysis membrane, 10 KDa) between CFE and silver nitrate solution. Protein extract was placed in membrane which was dipped in a beaker containing silver nitrate solution. Beaker was kept in dark and after 72 h dark brown colour was observed in membrane but not in surrounding solution. This indicates that the size of macromolecule required for nanoparticle synthesis is greater than 10 KDa. Controlled reaction shows development of dark brown colour indicative of AgNPs formation. 

Figure 4(c) shows the effect of cofactor on nanoparticle synthesis. Dark brown colour was developed in reaction mixture containing exogenously added cofactor, while colour intensity was less in flask without cofactor. Control containing only cofactor did not show any colour change. This proves the dependence of possible enzyme on cofactor and that the reaction is a redox reaction. 

**Figure 5:**
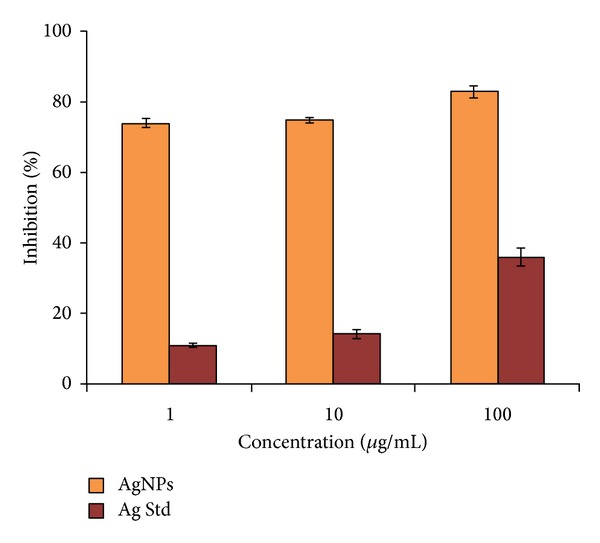
In vitro anticancer activities of silver nanoparticles synthesized by cell free extract of *S. boulardii*.

Anticancer activity of silver nanoparticles was evaluated on MCF-7 cells in comparison to ionic silver salt (Figure 5). Silver nanoparticles at very low concentration showed very high activity on MCF-7 cells, showing almost 80% inhibition at this stage also. At higher concentration (10–100 *μ*g/mL), no significant difference in inhibition of cancer cells was observed with silver nanoparticles. On the other hand, silver ions were able to achieve less than half of this even at 100 *μ*g/mL of concentration. This indicates that the IC_50_ value for the silver nanoparticles synthesized by *S. boulardii* whole cells is less than 10 *μ*g/mL.

## 7. Conclusion 

There are many reports that describe methods making use of microbes for the synthesis of nanoparticles [29–32] as an alternative to cost-ineffective and toxic procedures [1, 2]. The present method describes the first time use of cell free extract of *S. boulardii* for the synthesis of silver nanoparticles. Resulted nanoparticles were smaller in size, uniform, monodispersed in nature, and were synthesised very rapidly. This method does not require tedious downstream processing and it may be scaled up to develop a viable technology for the Ag-nanoparticle synthesis. Preliminary studies confirm the role of protein in the synthesis and stabilisation of nanoparticles. Anticancer activity on early stage breast cancer cell lines gave very promising results and this application may be valuable for future biomedical research.
